# Patterns of interest change in stack overflow

**DOI:** 10.1038/s41598-022-15724-3

**Published:** 2022-07-06

**Authors:** Chenbo Fu, Xinchen Yue, Bin Shen, Shanqing Yu, Yong Min

**Affiliations:** 1 Institute of Cyberspace Security, Zhejiang University of Technology, Hangzhou, 310023 China; 2 College of Information Engineering, Zhejiang University of Technology, Hangzhou, 310023 China; 3 Computational Communication Research Center, Beijing Normal University, Zhuhai, 519087 China; 4 School of Journalism and Communication, Beijing Normal University, Beijing, 100875 China

**Keywords:** Complex networks, Statistical physics, Computational science

## Abstract

Stack Overflow is currently the largest programming related question and answer community, containing multiple programming areas. The change of user’s interest is the micro-representation of the intersection of macro-knowledge and has been widely studied in scientific fields, such as literature data sets. However, there is still very little research for the general public, such as the question and answer community. Therefore, we analyze the interest changes of 2,307,720 users in Stack Overflow in this work. Specifically, we classify the tag network in the community, vectorize the topic of questions to quantify the user’s interest change patterns. Results show that the change pattern of user interest has the characteristic of a power-law distribution, which is different from the exponential distribution of scientists’ interest change, but they are all affected by three features, heterogeneity, recency and proximity. Furthermore, the relationship between users’ reputations and interest changes is negatively correlated, suggesting the importance of concentration, i.e., those who focus on specific areas are more likely to gain a higher reputation. In general, our work is a supplement to the public interest changes in science, and it can also help community managers better design recommendation algorithms and promote the healthy development of communities.

## Introduction

In recent years, benefiting from the ongoing process of datafication, more and more data are being collected and analyzed to discover human activity patterns^1–4^. Meanwhile, the constantly developing and cooperating of computer and social science prompt scientists to explore the essential features of these activities^5–8^, e.g., innovation. Yet, little is known about the underlying strategies of exploring knowledge. Interest drives humans to explore knowledge in different domains, resulting in different exhibit strategies and further affecting future success. Current research in science shows that interest shift patterns are the representations when people decide to adopt what kind of knowledge exploration strategy. For scientists, shifting in interest will affect their productivity and the investment received^9,10^. Furthermore, with the growth of their careers, the probability of scientists switching research fields will increase^10^. When scientists try to shift their interests, different exploration strategies result from the trade-off between stable productivity and creative innovation^11,12^. Basically, these strategies can be divided into two branches, conservative and radical strategies. Conservative strategy prefers to select existing and more traditional research directions. These directions may help scientists maintain stable productivity. However, when the knowledge exploration strategy within narrow boundaries, it is unlikely to be the source of the most fruitful ideas^12,13^. On the contrary, the radical strategy is prone to explore those new areas, bringing breakthrough results, praise, and success to scientists. Meanwhile, innovation and novel insights are more likely to source from exploring these areas^13–15^. Nevertheless, it is also a risky strategy, often associated with failure, reduced productivity, and the challenge of advancing ideas in the new academic world^16,17^. Despite the internal impact of researcher’s characteristics, the external academic environment also affects scientists’ interest shift and thus adopts different exploration strategies, such as the investors’ strategies^9,14^ and team collaboration^18–20^.

Persistence is a noble quality for humanity. In science, for those researchers who stick to their research fields, persistence will bring them rewards, for example, the lower probability of interest switching is often causing a higher number of citations^10^. However, the recent research shows that shift interest is necessary for success, e.g., those scientists who accumulate ideas in the exploration stage then concentrate on the focus research field in the development stage are more likely to lead to the “hot streak” emergence^21^. Moreover, there are many other factors that affect interest shift patterns, such as gender^22,23^, mobility^24–26^, reputation^27^, and mentor^28^. Although there are many influencing factors, the macro patterns of interest shifting are regular^29^, especially for scientists. Scientists have a high degree of regularity in their careers, e.g., Matthew effect in contributions^30^, random influence in publication^31,32^. However, there are still questions whether these patterns exist in the general public? To address this question, we study the interest change patterns of the general public in the Q &A community.

Question and Answering (Q &A) websites provide a channel for the general public to seek knowledge. Although they may not be as professional as scientific journals, they are useful and popular for the general public. Stack Exchange is one of the most popular Q &A websites, containing many Q &A communities in different particular domains. In these communities, Stack Overflow is the one for people seeking questions and answers in programming, providing the convenience to developers for catching up the rapid development of various skills and paradigms^33–37^. In order to attract more users, the Q &A communities introduce the gamification mechanism. This gamification mechanism motivates users to enhance their continuous learning ability and participation, e.g., reputation score, upvotes, downvotes, bounties, and badges^37–39^. In the community, reputation also implies the authority of users, a high reputation will encourage users to contribute knowledge to the community and regards as a badge of glory^40^. Because of the high contribution made by the Stack Overflow to the programming ecology, lots of researchers begin to study the collective intelligence behind the Q &A communities, such as the network in the community^41^, the unanswered questions^42^, low-quality posts^43^ and the quality of answers^44^. This platform also provides us the opportunity to study the interest change patterns of the general public, e.g., will the general public shift their interest to get a higher reputation? And the interest pattern study is also meaningful for the community managers to better adjust the recommendation system to attract more users.

To investigate the interest change patterns of the general public, we explore the interest change patterns in Stack Overflow, analyze the features that affect the patterns, more detailly, the main contributions of our work are as follows:

- Firstly, our study quantifies the changes of user interest in Stack Overflow and explores the overall pattern of interest changes.
- Secondly, our study find that changes in user interest are affected by three features: heterogeneity, recency, and proximity. The specific effects of these three features have been explored, and random experiments have been designed to prove it.
- Thirdly, we study the relationship between users’ interest and prestige changes and find that users with high prestige have lower interest changes.

The rest of the paper is organized as follows. “Methods” section introduces the dataset and presents the method to quantify the interest change. Then, the experiments and results are shown in “Results” section. Finally, “Discussion” section concludes with our works and future works.

## Methods

### Dataset

Our work is based on the publicly available dataset in Stack Exchange(https://archive.org/download/stackexchange), the main focus is Stack Overflow Q &A community, and the time frame spans Jul. 2008 to Sep. 2016. As summarized in Table 1, the dataset provides all the posts, including questions and answers, tags, posting dates, and **user reputation**. The statistical distributions of users and tags are shown in Supplementary Fig. S1. It can be seen that both distributions are subject to a power-law distribution, which means that most users tend to ask few questions (such as less than 50) and a large number of submitted tags are used only a few times (such as less than 50 times). In order to quantify the pattern of interest change of the individual, there need to be sufficient questions. Therefore, our work focus on the active users who asked more than 50 questions, totaling 31,303 users. Furthermore, **tags** are the words selected by the users to cover the question’s domain broadly. To make sure the tags represent the technical directions of questions, only tags that occur at least 50 times are focused, totaling 19,978 tags.

**Table 1:** Data description in stack overflow.

Objects	#
#Users	2,307,720
#Tags	46,264
Average questions per user	7.7
Average tags per question	2.97
Average life time per user (days)	526.33
#Users(>50 questions)	31,303
#Tags(>50 occurrences)	19,978

### Topic vector

Inspired by Jia’s work^29^, in this work, we analyze the sequence of user questions in Stack Overflow and quantitatively show how individual shift their interest focus over time. To capture the evolution of interest and systematically address the interest patterns of Q &A community users, we calculate each user’s topic vector. Furthermore, the question’s topic is abstracted to the tags. However, the tag is mainly determined by the poster, thus may cause custom labels that have never appeared before, which will result in too many tags. Thus, in order to further condense the topic, we construct the tag network. Specifically, the nodes represent the tags in the tag network, and the tags are connected if they co-occurrence in the same question. The tag network is then divided into communities by the Infomap algorithm^45^, an efficient discovery non-overlapping community algorithm based on information theory. Finally, this tag network is divided into 327 communities and about 100 communities with high attention (Supplementary Fig. S2), i.e., containing lots of tags. The characteristics of the tag network provided in Supplementary Tab. S1. Each community represents a **topic** or a main technical direction in the Stack Overflow.

**Figure 1:**
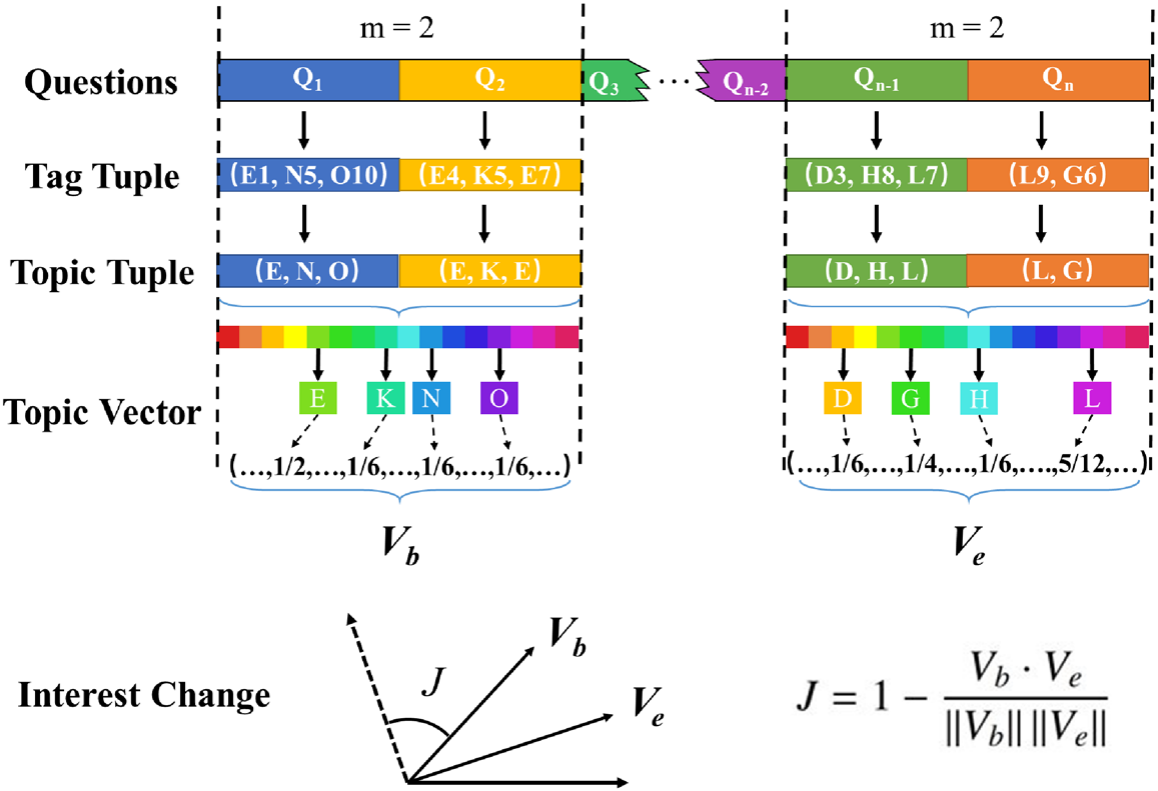
An example to calculate interest change $J (m=2)$. Firstly, the technical topic of the question is determined according to the question tags. Then, the initial topic vector $V_{b}$ and the final topic vector $V_{e}$ are generated based on the initial and final *m* questions. Finally, the interest change *J* is measured according to the complementary cosine similarity between topic vectors.

When a user submits a question $Q_i$, the corresponding tags constitute a **tag tuple**, e.g., (A1, B2, C3), where the capital letter indicates the topic to which the tag belongs. Further, the **topic tuple** can be represented by (A, B, C). Additionally, for a given set of questions submitted by a community user, the **topic vector** ${\textbf {V}} = (t_1, ... t_i, ... t_N)$ represents the user’s interest, ${\textbf {V}}\in R^N$, *N* is the number of topics in the Stack Overflow. Where $t_i = 0$ if the user has not submitted the *i*th topic, otherwise $t_i = \sum _{q=1}^{m}f_{i, Q_q}/m$, $f_{i, Q_q}$ is the normalized frequency of occurrence of the *i*th topic in the *q*th submitted question $Q_q$ and *m* is the number of questions in subsequence. As an example shown in Fig. 1, taking two consecutive questions as subsequence, e.g., ($Q_1$, $Q_2$) with $m = 2$, the tag tuples of the questions are (E1, N5, O10) and (E4, K5, E7) respectively, and the topic tuples are (E, N, O) and (E, K, E) respectively. Thus, the element value of topic E can calculate as $(1/3+2/3)/2=1/2$, because topic E appears once in $Q_1$ and twice in $Q_2$. The detailed definitions of bolded words are provided in Supplementary Note 1.

### Interest change

The user’s interest may change over time, thus, to quantify this pattern, our study takes the first and last *m* questions to characterize the interest change. Specifically, as shown in Fig. 1, the beginning topic vector $V_b$ and end topic vector $V_e$ are calculated through the first and last *m* questions. Then the interest change can quantify by the complementary cosine similarity as:

1 $$\begin{aligned} J=1-\frac{V_{b}\cdot V_{e}}{ \Vert V_{b}\Vert \Vert V_{e}\Vert }. \end{aligned}$$

Equation (1) captures the user’s interest change from individual activities in the Q &A community in a topic view. Extremely, if $J=0$, the beginning and end *m* questions share the same topic, which means the user’s interest never changes. Contrarily, if $J=1$, the beginning *m* questions’ topic is different from the last *m* questions, which means the user’s interest completely changes, in other words, the user no longer participates in the original topic of interest.

### Accordance statement

The dataset we used for Stack Overflow is publicly available(https://archive.org/download/stackexchange) and cc-by-sa 4.0 licensed. All methods were carried out in accordance with relevant guidelines and regulations.

## Results

**Figure 2:**
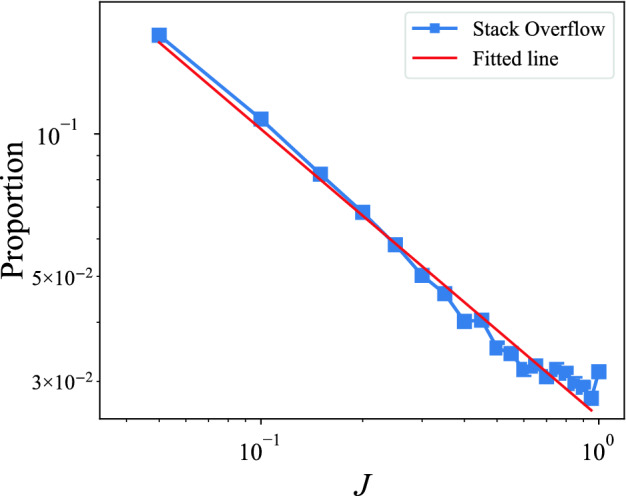
Distribution of interest change *J*. The blue dotted line is the proportion of *J*. We choose least squares regression to fit the data, and the fitted result is shown with the red line ($P \sim J^{-0.608}$), *P* is the proportion of *J*. The proportion decreases with the increase of *J* and can be well fitted by the power-law function.

To exhibit the overall scenery of the interest changes for the entire community, we plot the distribution of users’ interest change in the Stack Overflow. As shown in Fig. 2, this distribution follows a power-law distribution, which indicates that most Q &A users have little changes in their topic interests, however, there are still users who significantly switch their topic interests, albeit very rarely. Furthermore, it is interesting to find that the distribution in the Q &A community is quite different from the academic^29^, i.e., the distribution of research interest in the academic follows an exponential distribution but in Stack Overflow follows a power-law distribution. Compare with the academic field, the proportion of users with large *J* in Stack Overflow is higher. In order to characterize what affect the pattern of interest change in detail, our study investigates three features: heterogeneity, recency and proximity, and the corresponding experiments demonstrate as follows. Furthermore, in our experiment, $m=15$, however, the different values of *m* (such as $m= 5,10,20$) are also tested, and the power-law distribution characteristics of the *J* distribution remain unchanged.

### Heterogeneity

**Figure 3:**
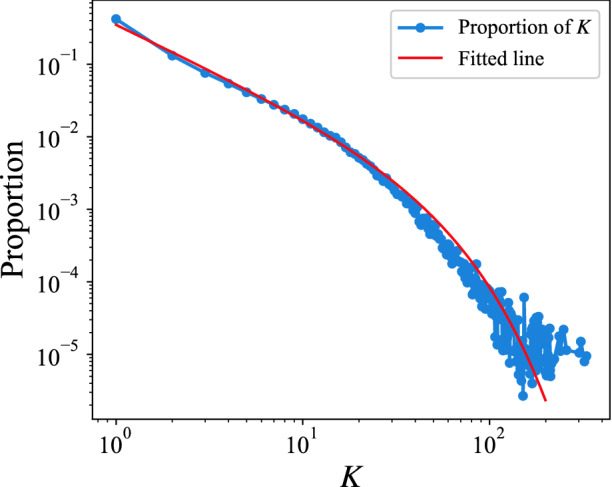
Frequency of topic tuple. *K* is the number of times that the same topic tuple occurs in a question sequence. The blue dotted line is the proportion of *K*. We choose least squares regression to fit the data, and the fitted result is shown with the red line ($P \sim K^{-1.22} \mathrm {e}^{-0.028 K}$), *P* is the proportion of *K*. The distribution shows a power-law distribution with exponential cutoff, which indicates the heterogeneity of interest change.

**Figure 4:**
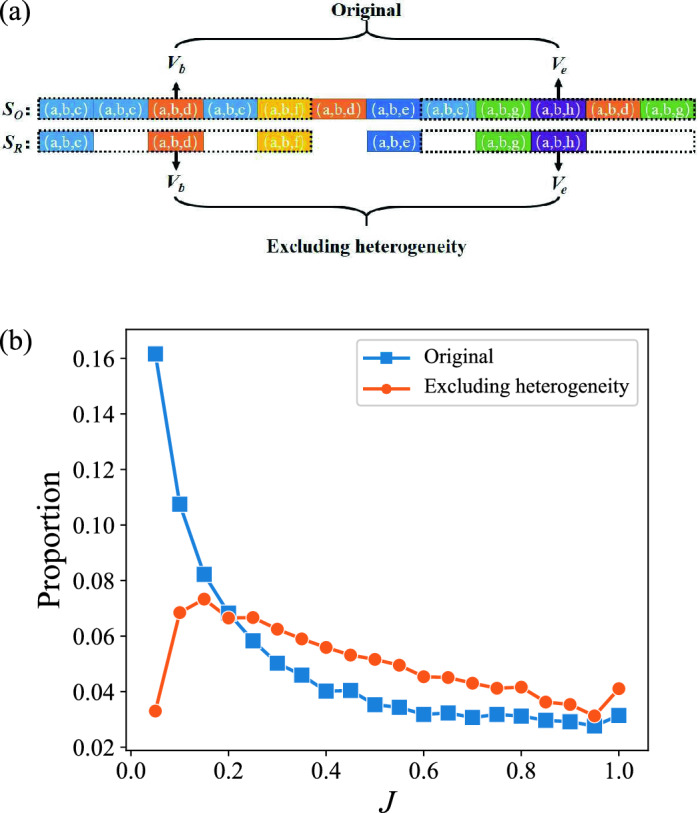
Heterogeneity of interest change. (**a**) Removal of heterogeneity. Only the topic tuple that appears for the first time is retained, to ensure that the user’s attention to each different domain is the same in the modified sequence. The removed questions are replaced with zero vectors. $S_O$ is the original sequence, and $S_R$ is the modified sequence. (**b**) The distribution of *J* after excluding heterogeneity. The blue line is *J* of the original data, and the orange line is the *J* of data after removing heterogeneity.

For an individual in the Q &A community, her attention to different topics may not be homogeneous, which means her interest range may contain the core interest subjects coexistence with the few other occasionally touched topics. For example, the mobile phone developer may use JAVA and Android tags and occasionally appears Windows tag. To verify this, we plot the frequency of topic tuples in Fig. 3. The power-law distribution clearly demonstrates the heterogeneity feature in the individuals’ interest topic. To further explore this feature, we remove the heterogeneity of the topic tuple sequence, i.e., only the topic tuples that appear for the first time are retained, and the remaining recurring topic tuples are replaced with zeros, thus the length of sequence does not change (Fig. 4a), then exhibit the comparison result in Fig. 4b. The difference in distribution is quite significant for the original and modified *J* distribution. The modified *J* distribution shows a sharply rising trend followed by a slowly falling, eliminating the original data’s power-law decrease. This phenomenon is similar in the academic field, that is, after removing heterogeneity, the proportion of people with small *J* decreases significantly in the academic field^29^. It implies that heterogeneity plays a role in limiting interest changes in both fields. The difference between the heterogeneity in academic publication and that in Stack Overflow is that the frequency of the number of questions with the same topic tuples submitted by user decreases slower than that of the papers with the same topic tuples published by scientist. Additionally, a jump occurs when $J=1$, which is mostly because of our way of removing the heterogeneity. The high repetition between the beginning and end topic tuples causes the smaller end topic vector. Extremely, if all the elements in the end topic tuples have appeared before, then $V_e=0$ and $J=1$.

### Recency

**Figure 5:**
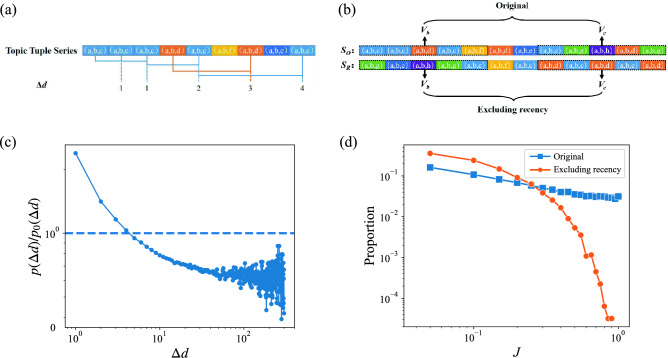
Recency of interest change. (**a**) An example to get the $\Delta {d}$ sequence. The number in the sequence represents the interval between the questions with same topic tuple, denoted as 0 if a topic tuple is the first occurrence. (**b**) Removal of recency. For the reshuffled sequence, randomly change the order of each question. $S_O$ is the original sequence, and $S_R$ is the modified sequence. (**c**) The ratio between the distribution of $\Delta {d}$ of real data $P(\Delta {d})$ and that of the reshuffled sequence $P_{0}(\Delta {d})$. The ratio implies that questions raised recently have had a greater impact on users than those raised long ago. (**d**) The distribution of *J* after excluding recency. The blue line is *J* of the original data, and the orange line is the *J* of data after removing recency.

The recency is the tendency to redo things similar to what has been done recently. To investigate this feature in the Stack Overflow, we focus on the distance between the topic tuples, denoting as $\Delta {d}$, which is defined as the number of different topic tuples between two identical topic tuples. Calculating $\Delta {d}$ on the entire topic tuples sequence, we can get the $\Delta {d}$ sequence, as the example shown in Fig. 5a. Then we construct a null model, which reshuffling the original topic tuple sequences of the user. For the question sequence, the length of the sequence is constant, but the order is shuffled (Fig. 5b). To compare the distribution $P(\Delta {d})$ with the reshuffled distribution $P_{0}(\Delta {d})$, we plot the distribution of ratio $P(\Delta {d})/P_{0}(\Delta {d})$ as function of $\Delta {d}$, as shown in Fig. 5c. It is found that the ratio declines as $\Delta {d}$ increases, which implies the Q &A community users tend to submit questions in the same domain as they have recently submitted, and rarely return to their original interest after turning to a new interest, prompting users to explore the new domains continually. Furthermore, the reshuffled model eliminates the power-law decrease observed in the original distribution and behaviors a steeper decrease with an exponential distribution from the view of interest change, as shown in Fig. 5d. The significant changes in the interest change distribution verify the recency feature does exist in the Q &A communities when users explore their interest. Compared to the academic field^29^, the trend of observed *J* distribution after excluding recency is similar in the small *J* range, the proportion of people with smaller *J* (near 0.2) is larger than the original distribution. This phenomenon implies that recency plays a similar role in increasing the proportion of *J* in both fields. However, excluding recency in Stack Overflow prompts the observed distribution from a power-law distribution to an exponential distribution, while in the academic publication, the distribution maintains exponential but decays steeper. As the form of distribution changes from power-law to exponential in Stack Overflow, the proportion of users with extremely large *J* decreases more significantly than in academic publication. Excluding recency changes the distribution of *J* from power-law to exponential in Stack Overflow but not in academic publication, which implies that recency affects the users more than scientists. To further illustrate the role of recency, we compare the proportion of the first *m* topic tuples repeated in the last *m* topic tuples before and after removing the recency of the sequence. The result shows that on average 17.74% of the topic tuples in the original data are repeated, and this proportion rises to 31.57% after removing the recency, indicating that the recency hinders people returning to the older direction of interest.

### Proximity

**Figure 6:**
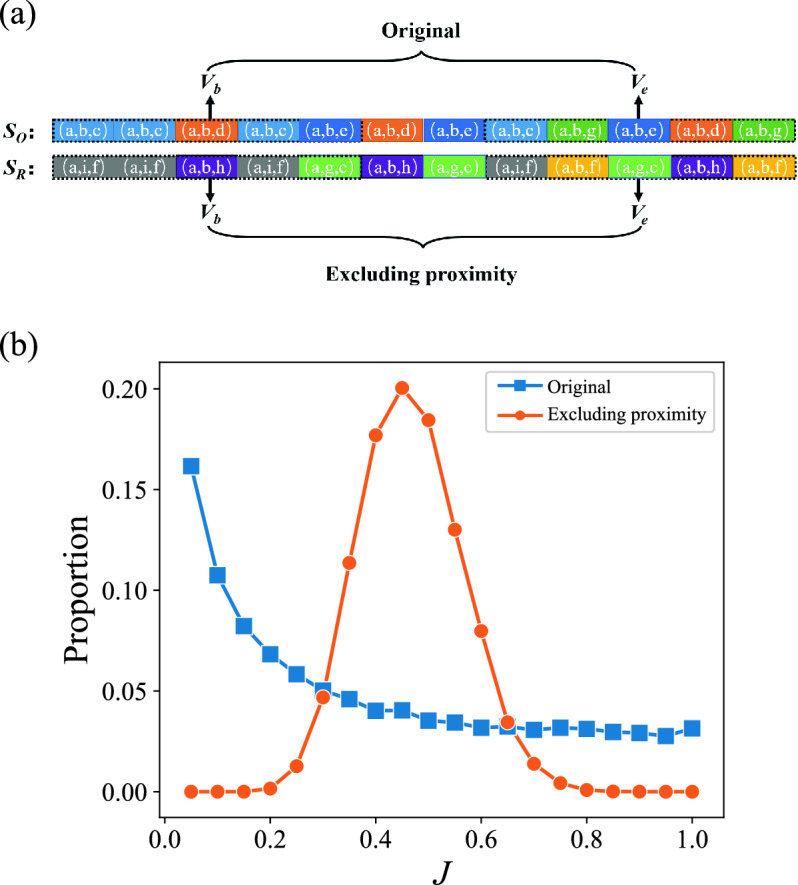
Proximity of interest change. (**a**) Removal of proximity. Replace each distinct topic tuple by randomly choosing a topic tuple in the topic tuple pool. The number and order of each topic tuple’s occurrences are unchanged. $S_O$ is the original sequence, and $S_R$ is the modified sequence. (**b**) The distribution of *J* after excluding proximity. The blue line is *J* of the original data, and the orange line is the *J* of data after removing proximity.

Unlike the recency feature describing the user’s interest pattern from a time perspective, the proximity feature studies the pattern from the topic geographic view. In the Q &A community, the proximity feature is reflected in the situation when users want to explore a new interest domain, the domain they chose is more similar to their current interest domain than a new field. To verify this, we focus on the proximity distance with the definition of interest change holds. Specially, we replace each distinct topic tuple by randomly choosing a topic tuple in the topic tuple pool which stores all topic tuples in the data, and keeps the length of the sequence not changed. It should be noted that, in the randomized sequence, the number of each topic tuple and the order that topic tuple is used are retained. For example, as shown in Fig. 6a, the original topic tuples sequence $S_O$ is “(a, b, c), (a, b, c), (a, b, d), (a, b, c), ...”, we replace (a, b, c) with (a, i, f) and (a, b, d) with (a, b, h), respectively. Where the topic tuples (a, i, f) and (a, b, h) are randomly chosen from the pool, which stores all topic tuples in the data. Finally, the modified sequence $S_R$ is “(a, i, f), (a, i, f), (a, b, h), (a, i, f), ...”, whose relative position of topic tuples has not changed. In this way, the modified sequence simulates that when the user changes their interest field, the new field has no relationship to the current field. The obtained distribution shows that excluding the proximity feature simultaneously reduces the proportion of users with small *J* ($J < 0.3$) and large *J* ($J > 0.7$), which fits Normal distribution $\mathcal {N}(\mu , \sigma ^{2}$) well (the value of chi-square is 0.0076, which is quite small), as shown in Fig. 6b, where $\mu$ is mean and $\sigma ^{2}$ is the variance. The phenomenon is different in the academic publication, after excluding the proximity, only the proportion of scientists with small *J* decreased^29^. The decreases in the proportion of users in Stack Overflow with small *J* and that of scientists with small *J* imply that proximity is one of the reasons for their interests to change slightly. However, proximity has different effects on different users in Stack Overflow. The decrease in the proportion of users with large *J* implies that the effect of proximity is also one of the reasons for their interests change vastly, they will explore fields that are less relevant to the initial field after being affected by proximity. In summary, the proportion both of small and large *J* in Stack Overflow is reduced after excluding proximity, which implies that proximity has the effect of limiting or promoting interest change, and the effect is different for different users.

### Reputation

**Figure 7:**
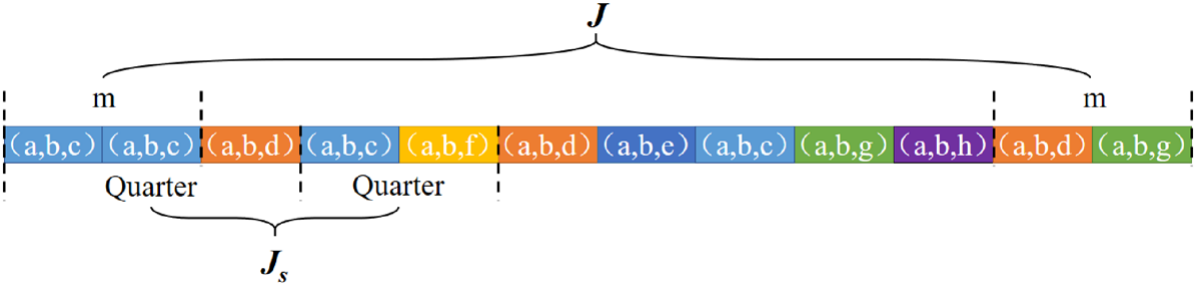
The difference between question selected, when calculate *J* and $J_s$. To calculate $J_s$, we use quarter window instead of *m* questions, e.g, in the first quarter, it contains three topic tuples, i.e., (a,b,c), (a,b,c) and (a,b,d), in the adjacent second quarter, it contains two topic tuples, i.e., (a,b,c) and (a,b,f).

**Figure 8:**
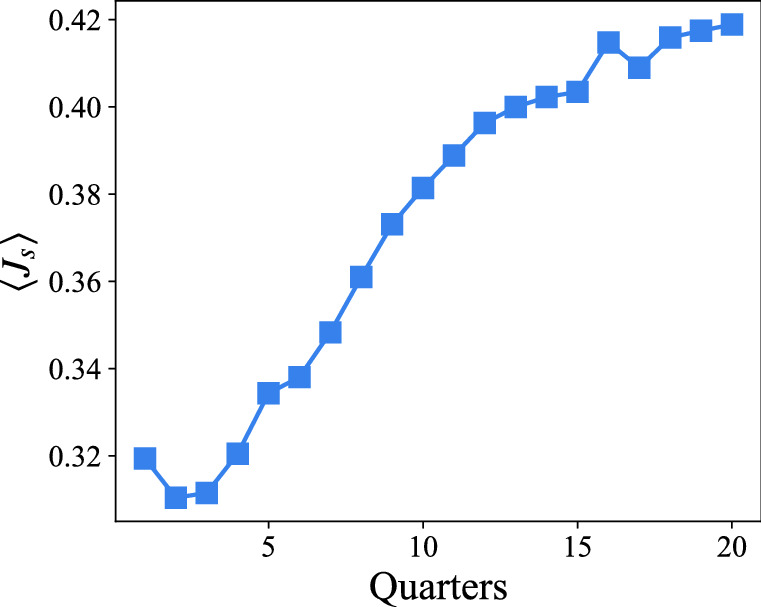
Relationship between time and $\langle J_s \rangle$. The average short-term interest change $\langle J_s \rangle$ is linearly correlated with time that user spend in the community.

**Figure 9:**
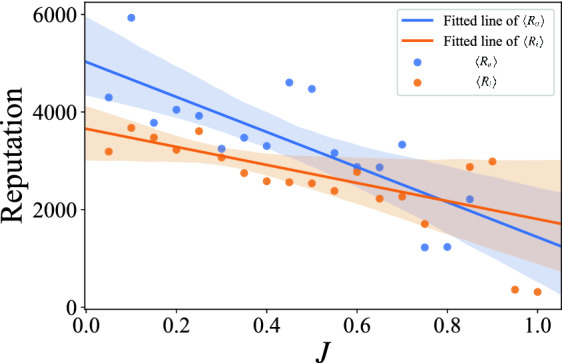
Relationship between user reputation and interest change. The blue dot represents the average reputation of active users with similar *J* values $\langle R_a \rangle$. The orange dot represents the average reputation of inactive users with similar *J* values $\langle R_i \rangle$. The solid lines are the fitted lines of reputation and *J*. The blue line is the fitted line of $\langle R_a \rangle$, and the orange line is the fitted line of $\langle R_i \rangle$. The shaded areas are the confidence interval (0.95). The coefficient of determination $R^2$ of active and inactive user is 0.594 and 0.261, respectively. In order to exclude the influence of career length on reputation, we only selected users whose career length is 40–60 quarters.

Scientists pay great attention to their researches quality and impact, they collaborate and earn reputations in academia^6,18,46,47^. Interestingly, reputation also prompted the Q &A community users to be more active in the community, e.g., submitting high-quality questions and answers quickly^48–50^. These phenomena trigger us to explore the relationship between reputation and user behavior on exploring interest. To do this, we first check users’ average short-term interest change quarterly. Specifically, denoting $J_s$ to quantify the short-term interest change of users. The calculation of $J_s$ is similar to *J*, but topic tuples in two consecutive quarter-time windows are used instead of the beginning and end topic tuples. The short-term interest change refers to the interest change between the questions in the adjacent two quarters. When calculating $J_s$, we use the quarter as the time window instead of *m* questions and calculate the topic vector, then calculate interest changes of adjacent quarters in the sequence as shown in Fig. 7. In order to calculate average short-term interest change $\langle J_s \rangle$ in *i*th quarter, we calculate all users’ $J_s$ in adjacent *i* and $i+1$ quarter, and normalized them with the number of users. The time users post their first question is chosen as the start point of the quarter-time window of each user. Figure 8 depicts the evolution of $\langle J_s \rangle$ over time, where the time window is selected as a quarter. The observed increasing trend indicates that users are accustomed to continuously switching interests. Scientists switch research fields for productivity, but it will negatively affect their influence^10^. Inspired by this phenomenon, we raise the question of how would users’ changing interests affect their reputation? To address this question, we study the relationship between reputation and *J* for active and inactive users, as shown in Fig. 9. The active users are selected if a user raises questions every month from the beginning to the end during the whole career, conversely, the user who has not asked a question for a month is considered as an inactive user. The figure shows that the interest change *J* negatively correlated with user reputation, whether active or inactive users. Furthermore, the reputation of active users is always higher than inactive users when interest change *J* is small. One plausible explanation is that exploring new domains is a risky strategy, not all explorations are fruitful. Continuous switching of interests may make individuals impossible to develop knowledge and capabilities in the focal domain. Furthermore, the reputation also will be attributed to users who continuously contribute in the same domain. This pattern underlines the importance of concentration and may not be a particular case of the general public when exploring their interests. Similar patterns are observed among the scientists, e.g., Ref.^10^ finds that the scientists with the high citations have the lower probability to change their research direction in their career periods.

## Discussion

In summary, our work studies the Q &A community user’s interest change patterns. Interestingly, our findings show that the user’s interest change follows a power-law distribution, which is entirely different from the research interest change distribution of the scientists (exponential distribution), indicating that users in the community are more inclined to exploration strategy. Compared to scientists, due to scientists’ characteristics, i.e., the long-term accumulation of discipline knowledge, scientists are more inclined to explore in the previous research stage and then concentrate on their current topics^10,21^. Despite this, the relationship between user interest change and reputation indicates that if users want to get a higher reputation in the Stack Overflow community, concentrating on the topic is still necessary. This phenomenon also highlights the difference between the general public and scientists in exploring knowledge strategies. Moreover, the user’s interest may shift to a new domain that is entirely different from the original over time, suggests that the community managers could consider the characteristics of user interest change when designing recommendation systems, e.g., pay more attention to the user’s current interests than consider all historical interest.

Furthermore, we study the three important features that significantly infer the observed distribution of interest change: heterogeneity, recency, and proximity. The heterogeneity makes user’s exploratory behavior more conservative on the Q &A community, while the recency feature has the opposite effect, it makes users explore new domains and result in a broader variety in interest change. The proximity feature prevents the interest change of users from presenting a Gaussian distribution. It increases the proportion of users with extreme interest change, e.g., the small-scale and large-scale interest change, which may be a reason for the power-law distribution of interest change. Moreover, the literature on research interest patterns of scientists^29^ also supports these trends of exploring knowledge. Furthermore, in this work, we only focus on the interest sequence, but ignore the timescale, which is another important feature. In future work, we will consider the timescale and investigate the explosive interest emerging in a short time. Additionally, in this work, we only consider the most straightforward community algorithm, however, the division result of the tag network may be influenced by the hypernym-hyponym relationship^51^. Thus, in the future, to make our division results more accurate, we will consider the hypernym-hyponym relationship in the division algorithm.

In general, our results provide a supplement to human interest research, showing how these features affect the patterns of interest in the Q &A communities and demonstrate the difference between the general public and scientific researchers in exploring knowledge. The current results would allow further expansion to uncover other interest behaviors in other communities as well as the relationships with different contribution types.

## Supplementary Information

Supplementary Information.
