# Supplementary material for: Patterns of interest change in stack overflow
Source: Sci Rep. 2022 Jul 6;12:11466. doi: 10.1038/s41598-022-15724-3 (PMC9259656; doi:10.1038/s41598-022-15724-3)
Supplement: Supplementary file 1 — Supplementary Information. [file 41598_2022_15724_MOESM1_ESM.pdf]

SUPPLEMENTARY INFORMATION

Patterns of Interest Change in Stack Overflow

Chenbo Fu1,2,*, Xinchen Yue1,2, Bin Shen1,2, Shanqing Yu1,2, and Yong Min3,4

[1]Institute of Cyberspace Security, Zhejiang University of Technology, Hangzhou, 310023, China
[2]College of Information Engineering, Zhejiang University of Technology, Hangzhou, 310023, China
[3]Computational Communication Research Center, Beijing Normal University, Zhuhai, 519087, China
[4]School of Journalism and Communication, Beijing Normal University, Beijing, 100875, China
*cbfu@zjut.edu.cn

Table of contents

**Heading**	**Topics**	**Page**
Supplementary Figure S1	Distribution of questions and tags	2
Supplementary Figure S2	Distribution of the number of tags	3
Supplementary Table S1	Characteristics of the tag network	4
Supplementary Note 1	Definitions	5

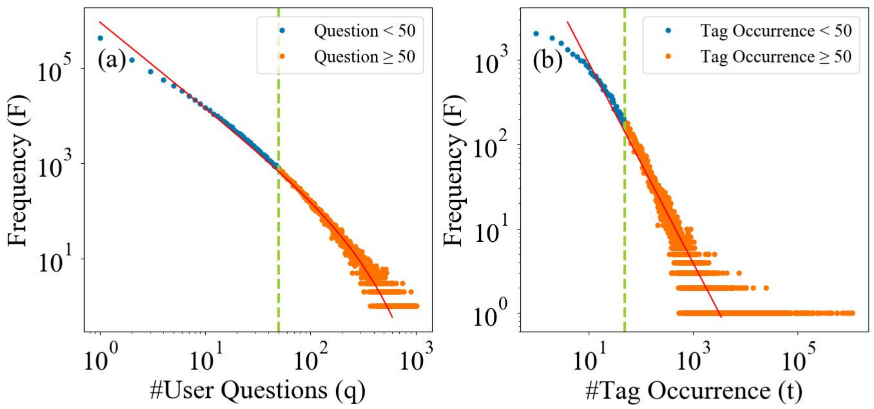

Supplementary Figure S1. Distribution of questions and tags. (a) Distribution of users' questions number. The orange dots are the users with more than 50 questions, and the blue dots are the users with less than 50 questions. The fitted result is shown with the red line. The distribution shows a power-law distribution with a slight exponential cutoff ($F \sim q^{-1.79} \mathrm{e}^{-0.0047\mathrm{q}}$). Compare with the result in Ref. 29 (Fig. 4(d)), our question exponent (1.79) is larger than the paper exponent (0.41), which implies the frequency of general public submit questions decreases faster than the scientist publish papers. This may be because although both systems are driven by interest, scientific exploration is a long process compared to asking questions, thus, the production distribution of science will not be as extreme as the Q&A community. To ensure there are enough questions to analyze the users' interest change, only user who has asked more than 50 questions are selected. (b) Tag occurrence number distribution. The orange dot is the tag which appear more than 50 times, and the blue dot is the tag which appear less than 50 times. The fitted result is shown with the red line. The distribution shows a power-law distribution ($F \sim t^{-1.19}$). The distribution shows a power-law distribution and obviously fat tail characteristics. The fat tail means that there have a few super popular tags, which may be due to the Matthew effect, i.e., the more popular the tag is, the more likely it will be used. Furthermore, many tags appear only a few times. It may be because users can create their own tags, but most users disapprove of such tags. To exclude very unpopular tags, we select only those that appear more than 50 times.

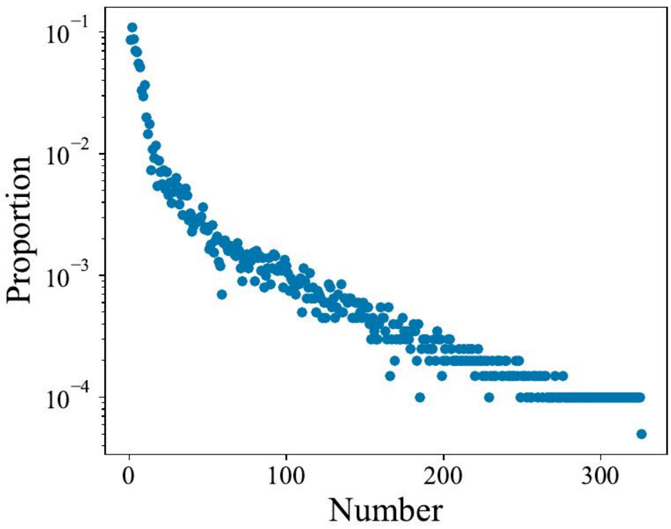

Supplementary Figure S2. The distribution of the number of tags in each topic after the community detection.

**Num of nodes**	19,978
**Num of edges**	840,502
**Average degree**	86.815
**Network diameter**	5.0
**Average path length**	2.384

Supplementary Table S1. Characteristics of the tag network.

In order to divide the community of tags so as to extract the topics of tags, we construct the tag network. The tag network uses tags as nodes. If two tags appear in the same question, an edge is added between the two tags. To help readers better understand our article, we present some basic characteristics of the tag network in Tab. S1.

Supplementary Note 1

Definitions. The words “tag”, “tag tuple”, “topic”, “topic tuple”, “topic vector” and “user reputation” have been used in the main text, but we only give them a rough explanation. To help readers better understand these words, we have provided the detailed definitions of these words.

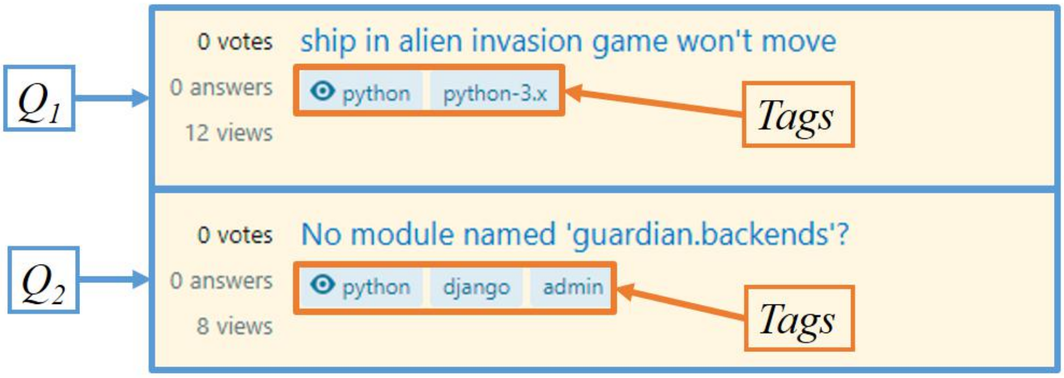

Supplementary Figure S3. Two questions in Stack Overflow. Blue boxes indicate questions Q_1 and Q_2. Orange boxes indicate tags of the questions.

Tag: A keyword or label that categorizes users’ question with other, similar questions, e.g., as shown in Fig. S3, “python” and “python-3.x” are tags of question Q_1, “python”, “django” and “admin” are tags of question Q_2.

Tag tuple: A tuple consisting of all tags in question, each question have one tag tuple, e.g., “{ python, python-3.x }” is the tag tuple of Q_1, “{ python, django, admin }” is the tag tuple of Q_2.

Topic: According to the result of community division of the tag network, each community has a topic, and tags in the same community have the same topic, e.g., tags “python” and “python-3.x” are in the same community A, thus they have the same topic “A”; “django” and “admin” are in different communities D and F, respectively, thus “django” has the topic “D” and “admin” have the topioc “F”.

Topic tuple: Replace the tag in the tag tuple with topic to which the tag belongs, e.g., the topic tuple of Q_1 is “{A, A}” transformed from tag tuple “{ python, python-3.x }”; the topic tuple of Q_2 is “{A, D, E}” transformed from tag tuple “{ python, django, admin }”.

Topic vector: Topic vector $V=(t_1, \ldots\ t_i, \ldots\ t_N)$ quantifies the technical fields involved in m questions in the form of a vector calculated according to the topic tuples, which represent users' direction of interest when they ask these m questions. Where N is the number of topics in the Stack Overflow, the element in the topic vector is calculated by following equation:

$$t_i = \begin{cases} 0\,, & \text{topic } i \text{ does not appear in topic tuples} \\ \sum_{q=1}^{m} f_{i,Q_q}/m\,, & \text{topic } i \text{ appears in topic tuples} \end{cases} \quad \text{(Eq. S1)}$$

where t_i is the element value of the topic i, f_{i,Q_q} is the normalized frequency of occurrence of the topic i in the topic tuple of question Q_q. Take Q_1 and Q_2 in Supplementary Fig. S3 as an example, in this situation, m is 2, the topic tuples are "{A, A}" and "{A, D, E}", respectively. Then the topic vector of these two questions is {2/3, …, 1/6, …, 1/6, …}, where the element of the vector is calculated by Eq. S1. For example, the first element is the value of topic A, the normalized frequency in the first topic tuple is $f_{A,Q_1} = 2/2$ and in the second topic tuple is $f_{A,Q_2} = 1/3$, the denominator is the number of topics in the topic tuple. Finally, the $t_A = (f_{A,Q_1} + f_{A,Q_2})/m = 2/3$.

User reputation: User reputation is a score proposed by the Stack Overflow community, which roughly measures how much the community trusts the user. With the growth of reputation, the community will give users more privileges, and the most basic way to gain reputation is to ask good questions and provide helpful answers. In this work, we directly use the user reputation provided by the community.
